# The Capacity of Magnesium to Induce Osteoclast Differentiation Is Greatly Enhanced by the Presence of Zoledronate

**DOI:** 10.3390/biology12101297

**Published:** 2023-09-29

**Authors:** Silvia Ricchiuto, Rossella Palumbo, Francesca Lami, Francesca Gavioli, Lorenzo Caselli, Monica Montanari, Vincenzo Zappavigna, Alexandre Anesi, Tommaso Zanocco-Marani, Alexis Grande

**Affiliations:** 1 Department of Biomedical, Metabolic and Neural Sciences, University of Modena and Reggio Emilia, 41125 Modena, Italy; 255698@studenti.unimore.it (S.R.);; 2 Department of Life Sciences, University of Modena and Reggio Emilia, 41125 Modena, Italy; 3 Department of Medical and Surgical Sciences for Children & Adults, University of Modena and Reggio Emilia, 41124 Modena, Italy

**Keywords:** Bisphosphonates, zoledronate, ONJ, osteoclasts, magnesium, differentiation

## Abstract

**Simple Summary:**

A number of skeletal disorders, all characterized by a metabolic or neoplastic loss of bone tissue, are cured with drugs called Bisphosphonates (BPs), which exert their therapeutic effect by suppressing cells named osteoclasts, normally mediating bone resorption. Unfortunately, these drugs can also provoke a dangerous side effect known as osteonecrosis of the jaw (ONJ), a bone infection localized in the oral cavity and characterized by gingival ulceration, sometimes accompanied by suppuration and pain. This condition, occasionally arising spontaneously, is more often started by a tooth extraction. The reduced number of osteoclasts, determined by BPs, is thought to favor the bacterial invasion of healthy bone and the incapacity to eliminate infected bone, that are in turn responsible for the appearance of ONJ. Here we show that Magnesium, used for decades as dietary supplement, can invert the effect of BPs, transforming them, through a sort of paradox effect, into powerful activators of osteoclast production. These results suggest that Magnesium might be used in a topical approach aimed to cure or prevent ONJ. Notably, the capacity of Magnesium to activate osteoclast production was even observed in absence of BPs, suggesting its application also in ONJ forms caused by agents distinct to BPs.

**Abstract:**

Bisphosphonates (BPs) are successfully used to cure a number of diseases characterized by a metabolic reduction in bone density, such as Osteoporosis, or a neoplastic destruction of bone tissue, such as multiple myeloma and bone metastases. These drugs exert their therapeutic effect by causing a systemic osteoclast depletion that, in turn, is responsible for reduced bone resorption. Unfortunately, in addition to their beneficial activity, BPs can also determine a frightening side effect known as osteonecrosis of the jaw (ONJ). It is generally believed that the inability of osteoclasts to dispose of inflamed/necrotic bone represents the main physiopathological aspect of ONJ. In principle, a therapeutic strategy able to elicit a local re-activation of osteoclast production could counteract ONJ and promote the healing of its lesions. Using an experimental model of Vitamin D3-dependent osteoclastogenesis, we have previously demonstrated that Magnesium is a powerful inducer of osteoclast differentiation. Here we show that, surprisingly, this effect is greatly enhanced by the presence of Zoledronate, chosen for our study because it is the most effective and dangerous of the BPs. This finding allows us to hypothesize that Magnesium might play an important role in the topical therapy of ONJ.

## 1. Introduction

Several diseases give rise to a metabolic reduction in bone density, such as is, for example, observed in osteoporosis, or a neoplastic destruction of bone tissue, which is detected in multiple myeloma and bone metastases of solid tumors. All these conditions can be cured using a class of drugs, defined Bisphosphonates (BPs), that counteract the loss of bone tissue by inhibiting the function of osteoclasts, i.e., the cells normally mediating bone resorption [1,2]. It has to be pointed out that, in addition to their anti-resorptive effect, BPs are also endowed with an intrinsic anti-tumor activity allowing them to directly limit the proliferation of malignant cells [3,4]. The practical consequences of a treatment with BPs are thus represented, in general, by a reduction in bone pains and a lower risk of developing bone fractures that, in the specific case of bone tumors, can also be accompanied by a slowdown of disease progression. The denomination of BPs reflects the presence of two phosphate groups in their molecule, rendering them structurally similar to Pyrophosphate (PP). Thanks to this feature, once administered to the patient, BPs are preferentially delivered to the bone tissue, where they bind to the hydroxyapatite crystals of its extra-cellular matrix [5]. In this location, BPs accumulate at very high concentrations, persisting there for long periods of time, even for the entire life of the patient. When bone is resorbed, BPs are released and internalized in great amounts by osteoclasts, causing their suppression, which can take place through different mechanisms, depending on the category they belong to. In fact, based on the presence of nitrogen in their molecule, BPs are divided into two groups: Nitrogen-containing (N-BPs) and Non-Nitrogen-containing (Non-N-BPs). N-BPs, like Zoledronate and Pamidronate, act inhibiting the farnesylation of G proteins like Ras, which is required for the intra-cellular transduction of tyrosine-kinase receptor signaling [6]. This effect is reached through competition with a set of PP-bound lipids mediating such reaction. Non-N-BPs, like Clodronate and Etidronate, instead, act replacing PP in the ATP molecule, thus impairing its hydrolysis and the contextual release of energy [7]. From a biological point of view, it is therefore evident that both mechanisms are based on the capacity of BPs to mimic PP structure, finally leading to a common effect that is always represented by the apoptosis of osteoclasts. On the other hand, among the more clinical aspects diversifying the two BP categories, it is worth mentioning that N-BPs are more powerful and more prevalently used in neoplastic bone disorders whereas Non-N-BPs are less active and are more substantially used in metabolic bone diseases [8]. Unfortunately, despite their beneficial activity, BPs can also determine a frightening side effect named osteonecrosis of the jaw (ONJ) [9]. This condition appears, as a rule, with a gingival ulceration that is less frequently associated with suppuration and pain [10,11,12]. Although ONJ can arise spontaneously, it is more often triggered by a surgical intervention, typically a tooth extraction [13,14]. According to the current view, the primary event causing ONJ is the invasion of alveolar bone by bacteria deriving from the oral cavity. This circumstance is then responsible for a situation of infection, inflammation, and necrosis of the interested site [15,16]. It is conceivable that the osteoclast depletion, induced by BP treatment, favors both the entry of bacteria into the initially healthy bone and then the inability to eliminate the damaged bone [17]. As expected, the probability of undergoing ONJ correlates with the efficacy of the responsible BP and, on this basis, it is higher with N-BPs as compared to Non-N-BPs. With Zoledronate, the most powerful but also the most hazardous among the BPs, the risk of developing ONJ was estimated at 3% and 21%, respectively, after two and four years of treatment [18]. To date, there is no an available medical therapy to cure this condition that can be exclusively approached with a surgical treatment consisting of the removal of necrotic bone [19,20,21,22]. The local re-activation of osteoclast function, inhibited by the systemic therapy with BPs might, in theory, cure and, perhaps, also prevent ONJ [23]. Using an experimental model of Vitamin D3-dependent osteoclastogenesis [24], based on the U937 cell line [25], we have previously demonstrated that, when employed at supra-physiological concentrations, Magnesium acts as a powerful inducer of osteoclast differentiation [26]. Operating under the same conditions, we tried here to clarify whether this capacity is also maintained in the presence of Zoledronate, selected among the other BPs, to conduct our study, in light of its peculiar biological and pharmacological properties [18,27]. The results obtained were really surprising since they not only demonstrated the inability of Zoledronate to contrast the osteoclast differentiation effect promoted by high levels of Magnesium, but they also provide evidence that the latter was strongly potentiated by the presence of the former.

## 2. Materials and Methods

### 2.1. Cell Cultures

The U937 cell line was obtained from the American Type Culture Collection (ATCC; Rockville, MD, USA) and cultured at 37 °C, 5% CO_2_ in a RPMI1640 medium (Euroclone, Devon, UK), supplemented with 10% heat inactivated foetal bovine serum (FBS) (Sigma-Aldrich, St. Louis, MO, USA) and 1 mM L-Glutamine (Euroclone). All experiments aiming to assess the proliferation activity and the extent of apoptosis in U937 cells were performed under the standard maintenance conditions described so far.

#### 2.1.1. Osteoclast Differentiation

When required, osteoclast differentiation of U937 cells was induced by a 2-day stimulation with 48 nM Phorbol 12-Myristate 13-Acetate (PMA) (Sigma-Aldrich), followed by a PBS rinse, aiming to remove non-adhering undifferentiated cells, and then by a 3-day stimulation with 10^−8^ M 1α, 25 di-hydroxy Vitamin D3 (VD3) (Sigma-Aldrich) [26].

#### 2.1.2. Pharmacological Treatments

Zoledronic acid (synonymous with Zoledronate) (ZA) and Magnesium Chloride (MgCl_2_) were both purchased from Sigma-Aldrich, dissolved in sterile water and added to cell cultures of both proliferating and differentiated U937 cells, at the concentrations that are indicated in detail in the Results section.

#### 2.1.3. Cell Counts

Cell counts were carried out on samples diluted with an equal volume of Trypan Blue (Sigma), using a TC20 automated cell counter (Bio-rad, Munich, Germany) and considering living cells in the size range between 8 and 18 μm.

#### 2.1.4. Morphological Analysis

Morphological analysis was conducted with a digital microscope Evos M500 Imaging System (Thermo Fisher Scientific, Waltham, MA, USA) on cell samples that had been previously subjected to cyto-centrifugation and May–Grunwald–Giemsa staining.

### 2.2. Flow Cytometry

Flow cytometry analysis was performed using an Attune NxT flow cytometer (Thermo Fisher Scientific, Waltham, MA, USA) to assess cell cycle, apoptosis, and differentiation.

#### 2.2.1. Cell Cycle and Apoptosis Analysis

Cell cycle and apoptosis were evaluated by flow cytometry analysis upon Propidium Iodide (PI) staining [28]. This procedure allows the identification of apoptotic cells and cells in the various phases of the cell cycle, based on the amount of dye incorporated into labeled cells and bound to their genomic DNA. Samples undergoing this analysis were prepared for flow cytometry by a 30 min incubation at 4 °C in Nicoletti’s solution, containing 20 μg/mL PI, 0.1% Triton X-100, 0.1% Tri-sodium citrate, all provided by Sigma and dissolved in distilled water.

#### 2.2.2. Immune Phenotype Analysis

Differentiation was assessed by flow cytometry analysis of immune phenotype, using the CD11b antigen as a marker to estimate its entity [29]. To this aim, flow cytometry was preceded by an incubation of samples for a 30 min at 4 °C with phycoerythrin-conjugated (PE) mouse anti-human CD11b Monoclonal Antibody (MoAb-) (Miltenyi Biotec, Auburn, CA, USA), in PBS containing 5% FCS and 1% FcR blocking reagent (Miltenyi). At the end of labeling, cells were washed twice and finally re-suspended with PBS.

### 2.3. Molecular Analysis

Analysis of gene expression was conducted, at the mRNA level, using the Quantitative Real-Time Polymerase Chain Reaction (QRT-PCR) method, based on the Taqman procedure [30]. To be accomplished, this reaction required the extraction of RNA from each cell sample, and its reverse transcription (RT) into complementary DNA (cDNA).

#### 2.3.1. RNA Extraction

Total RNA was extracted using the Qiagen RNeasy plus mini kit (Qiagen, Valencia, CA, USA) and, once purified, its concentration was assessed using a NanoDrop 1000 spectrophotometer (Thermo Fisher Scientific).

#### 2.3.2. Reverse Transcription

A 200 ng amount of each RNA sample was subjected to RT with the HiScript III RT SuperMix for qPCR (Vazyme Biotech, Nanjing, China), that was used according to the manufacturer’s instructions, obtaining, in this way, the corresponding cDNA.

#### 2.3.3. QRT-PCR Analysis

Amplification was performed by adding to the cDNA sample the Taqman Gene Expression Master Mix (Thermo Fisher Scientific) and the proper Taqman Gene Expression Assays (Thermo Fisher Scientific), listed below together with their gene symbol and catalogue numbers. The QRT-PCR reaction was run in triplicate in a Light Cycler 480 detection system (Roche Diagnostics, Mannheim, Germany) thermal cycler. Normalization of signals was obtained using the Glyceraldehyde-3-Phosphate Dehydrogenase (GAPDH, Hs02786624_m1) mRNA as an endogenous control. Statistical analysis of QRT-PCR results was conducted using the 2^−ΔΔCt^ method, which calculates the relative changes in gene expression of the considered target mRNA normalized to the endogenous control and related to a calibrator sample. The values obtained were represented in terms of relative quantity (RQ) of mRNA level variations.

##### Analysis of Cell Cycle-Related Genes

This analysis was carried out performing the QRT-PCR reaction with the following reagents: D3 cyclin (CCND3; Hs01017690_g1), Cyclin Dependent Kinase Inhibitor 1 A (CDKN1A, also known as p21; Hs00355782_m1)

##### Analysis of Differentiation-Related Genes

This analysis was carried out performing the QRT-PCR with the following reagents: Nuclear Factor of Activated T cells 1 (NFATC1; Hs00542678_m1), Dendritic Cell Specific Transmembrane protein 1 (DCST1; Hs00984780_m1), Acid Phosphatase 5 (ACP5, also known as Tartrate Resistant Acid Phosphatase or TRAP; Hs00356261_m1), Cathepsin K (CTSK; Hs00166156_m1), Matrix Metallo Proteinase 9 (MMP9; Hs00234579_m1), Musculo—Aponeurotic Fibrosarcoma oncogene homologue B (MAFB; Hs00534343_s1), Cluster Designation 14 (CD14; Hs02621496_s1), Cluster Designation 163 (CD163; Hs00174705_m1).

### 2.4. Statistical Analysis

All experiments were repeated at least three times and the results were presented as mean ± S.E.M. values. Pairwise comparisons were carried out using the Student’s *t*-test, whereas multiple comparisons were performed with the Anova procedure. The results of the statistical analysis were considered significant when exhibiting *p*-values ≤ 0.05 and highly statistically significant when exhibiting *p*-values ≤ 0.01 and are reported in the Results section or in Table 1.

## 3. Results

### 3.1. Proliferative and Apoptotic Effects Determined on Cycling U937 Cells by Scalar Concentrations of Zoledronate

To assess the biological effects determined by ZA on proliferation and apoptosis, U937 cells were treated for up to four days with scalar concentrations of this drug ranging from 0 to 100 μM and then subjected to a cell count, flow cytometry analysis upon PI staining, and QRT-PCR analysis of D3 cyclin and p21 mRNA expression. Statistical analysis was carried out by a Student’s *t*-test, performed on pairwise comparisons between untreated control cells and cells treated with each tested ZA concentration.

**Figure 1:**
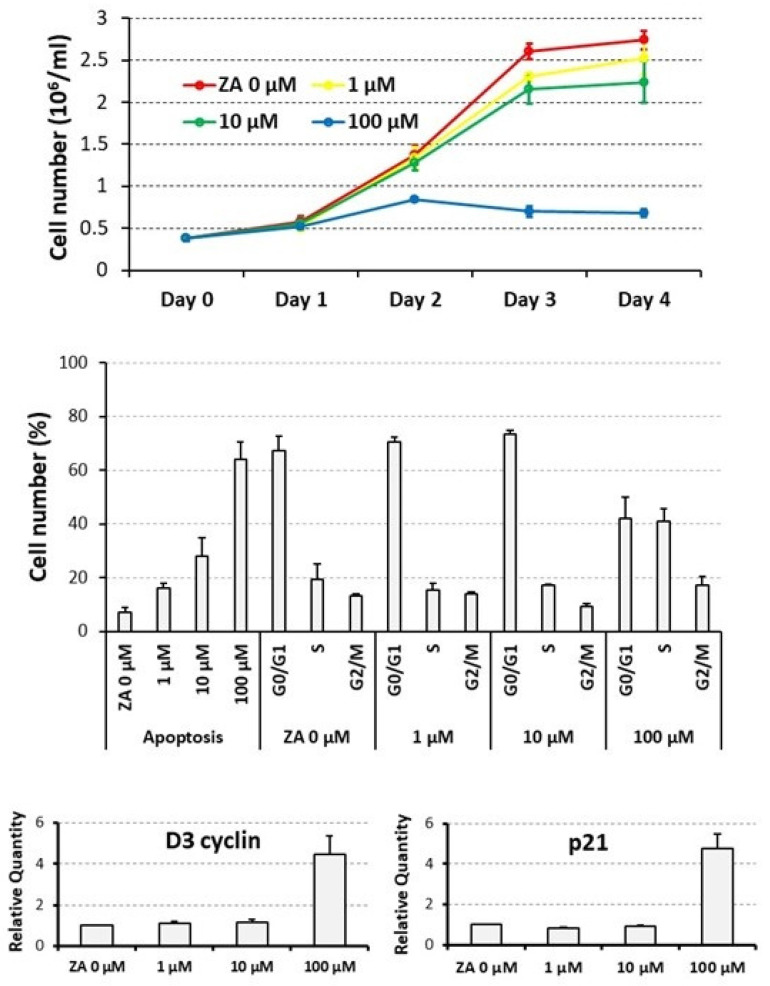
Proliferative and apoptotic effects exerted by scalar concentrations of Zoledronate on U937 cells. U937 cells underwent treatment with scalar concentrations of Zoledronate (ZA), ranging from 0 to 100 μM for up to 4 days. The (**upper panel**) shows the results of a daily cell count represented as a growth curve. The histogram of the (**middle panel**) depicts the percentage of apoptotic cells, and cells distributed in the various cell cycle phases, as assessed by flow cytometry analysis performed at day 4 of the experiment. The (**lower panel**) presents a histogram indicating the mRNA expression levels of D3 Cyclin and p21 genes, estimated by QRT-PCR analysis at day 4 of the experiment. Statistical analysis was always carried out by a Student’s *t*-test, performed on pairwise comparisons between the untreated control cells and cells treated with each tested ZA concentration.

A daily count revealed that U937 cells, treated with ZA, undergo a time and dose-dependent decrease in cell number (Figure 1, upper panel). In fact, at the end of the experiment, cell concentration, indicated as million/mL, averaged 2.7 ± 0.1 in the untreated control cells (0 μM) whereas the same parameter was 2.5 ± 0.2, 2.2 ± 0.2 and 0.7 ± 0.1 in samples, respectively, treated with 1, 10 and 100 μM ZA. Therefore, the reduction in cell number was remarkable with the highest drug concentration (100 μM), whereas it was quite limited with the lower ones (1 and 10 μM). Consistently, *p* values appeared highly significant for the former, already starting from the second day of treatment (0.005 at day 2, 0.0009 at day 3, 0.00003 at day 4), whereas they were not significant for the latter.

In agreement with the data presented so far, ZA treatment also elicited a dose-dependent apoptotic effect (Figure 1, middle panel), indicated by the fact that, always at day 4, the mean percentage of apoptotic cells resulted 7.2 ± 1.8 in untreated control cells (0 μM) and 16.1 ± 1.8, 27.8 ± 6.8 and 64.2 ± 6.5 in cells that had been treated with 1, 10 and 100 μM ZA, respectively. The corresponding *p* values were at least statistically significant (0.01, 0.03 and 0.007, respectively). Exposure to the same amounts of the investigated drug gave rise to a similar and not significant distribution in the various cell cycle phases, with the exception of the highest 100 μM ZA concentration, promoting a reduction in cells in the G0/G1 phase (41.9 ± 8.0% versus 67.1 ± 5.7% of control) and, in our opinion, a relative increase in the other cell cycle phases (for S phase, 40.9 ± 4.8% versus 19.5 ± 5.6%, respectively). This finding suggests that, in the considered sample, apoptosis was preferentially activated in the G1 phase of the cell cycle.

QRT-PCR analysis disclosed a 4.5 ± 0.9-fold increase in D3 cyclin transcript and a 4.8 ± 0.7-fold induction of p21 mRNA levels, that were exclusively observed with a 100 μM concentration of ZA, confirming the capacity of this drug to determine a growth arrest in the G1 phase of the cell cycle (Figure 1, lower panel). The mRNA expression of the two analyzed genes remained, on the contrary, unaffected with the lower ZA concentrations.

Taken together, these data indicate that ZA concentrations between 1 and 10 μM, also confirmed by other studies [31], exert the expected apoptotic effect on target cells and, at the same time, are well tolerated avoiding the strong growth arrest observed with the higher tested concentration. Consequently, the 10 μM concentration of ZA was chosen for the subsequent experiments.

### 3.2. Proliferative and Apoptotic Effects Determined on Cycling U937 Cells by a Supra-Physiological Concentration of MgCl_2_

We have previously demonstrated that 10 mM is the optimal concentration of MgCl_2_ favoring the osteoclast differentiation of U937 cells [26,27], but its effect on the proliferation and apoptosis of these cells has not been assessed before. For this reason, they were exposed to the mentioned concentration of MgCl_2_, under current cell culture conditions, and then analyzed as already explained for ZA treatment.

**Figure 2:**
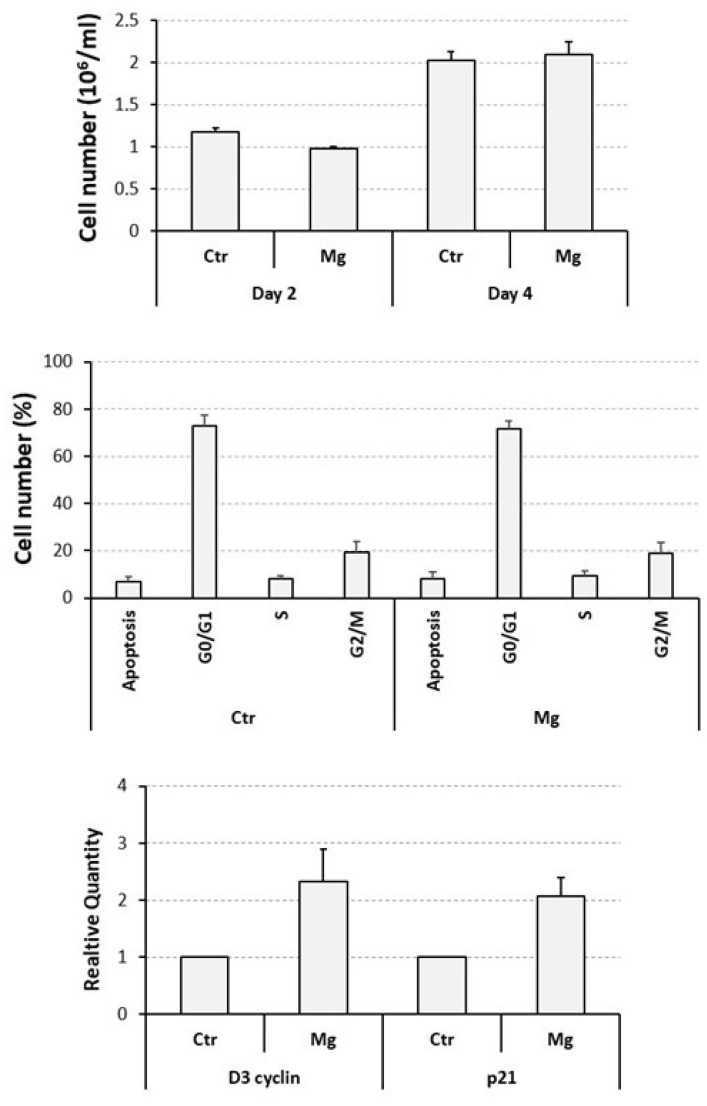
Proliferative and apoptotic effects exerted by a supra-physiological concentration of MgCl_2_ on U937 cells. U937 cells were exposed to a 10 mM concentration of MgCl_2_ and then subjected to a cell count, at 2-day intervals (**upper panel**), flow cytometry analysis, at day 4 of the experiment, to assess the percentage of apoptotic cells and cells distributed in the various cell cycle phases (**middle panel**), and QRT-PCR analysis, always at day 4 of the experiment, to evaluate the transcript levels of D3 cyclin and p21 genes (**lower panel**). All the results have been presented as histograms. Ctr, control, untreated cells; Mg, cells treated with MgCl_2_.

A cell count, performed at day 2 of the experiment and again indicated as millions/mL, put in evidence an about 15% statistically significant reduction in cell number, from 1.2 ± 0.1 of the untreated control to 1.0 ± 0.0 of the MgCl_2_ treated cells (*p* = 0.03) whereas, at day 4, the same treatment gave rise to an apparently inverted effect characterized by a 5% increase in cell number, from 2.0 ± 0.1 to 2.1 ± 0.2, respectively, although statistically not significant (Figure 2, upper panel). At the same time point, flow cytometry examination revealed similar and statistically not significant mean percentage values of apoptotic cells (8.1 ± 2.8 in MgCl_2_ treated cells versus 6.8 ± 2.1 of untreated control cells) and proliferating cells in the various cell cycle phases (respectively: 71.6 ± 3.2 versus 72.7 ± 4.9, for G0/G1, 9.6 ± 1.8 versus 8.0 ± 1.6, for S, 18.9 ± 4.6 versus 19.4 ± 4.6, for G2/M) (Figure 2, middle panel).

QRT-PCR analysis disclosed a modest but statistically significant upregulation of the mRNA expression of both the D3 cyclin and the p21 genes, respectively, undergoing a 2.3 ± 0.1 (*p* = 0.05)- and a 2.1 ± 0.3 (*p* = 0.03)-fold increase in response to MgCl_2_ treatment (Figure 2, lower panel).

These data globally indicate that MgCl_2_ is able to determine a weak and transitory inhibition of the proliferation of U937 cells. This effect can probably be interpreted as a delayed cell cycle entry and/or a prolonged doubling time. However, a 10 mM concentration of the considered compound appeared to be overall well tolerated by cells and suitable for further experimentation.

### 3.3. Capacity of MgCl_2_ and ZA to Modulate the Osteoclast Differentiation of U937 Cells Induced by Stimulation with Phorbol Esters and Vitamin D3

To assess the effect determined by MgCl_2_ and ZA on osteoclast differentiation, we adopted an experimental model of VD3-dependent osteoclastogenesis, already used in the past in our laboratory, and consisting of U937 cells undergoing an initial 2-day stimulation with 48 nM PMA followed by a 3-day stimulation with 10^−8^ M VD3 [25,26]. Treatment with 10 mM MgCl_2_ and 10 μM ZA was carried out, singly or in combination, for the entire stimulation period using untreated cells as a control. At the end of the cell culture, all samples were subjected to molecular, immunophenotype, and morphological analysis.

**Figure 3:**
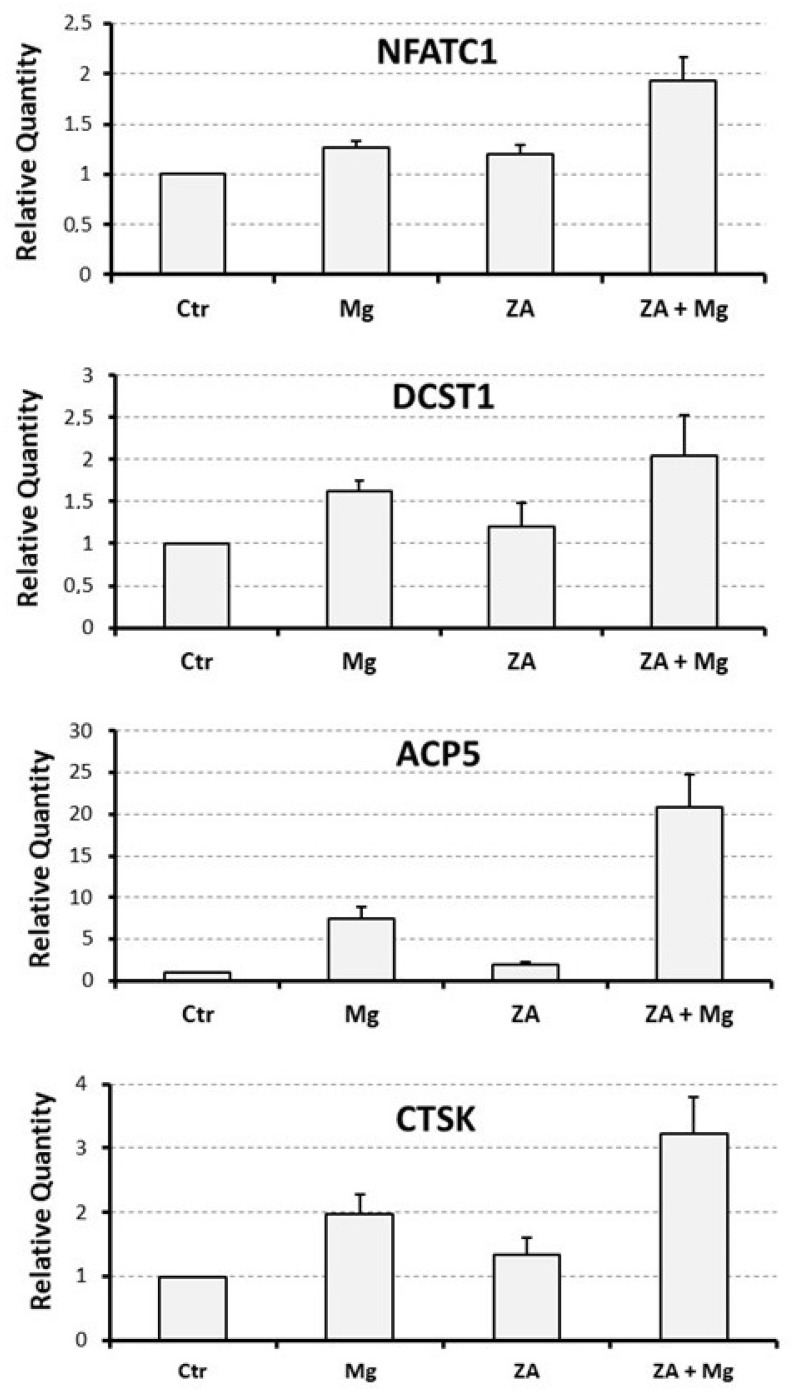
Effect determined by treatment with MgCl_2_ and ZA on the mRNA expression of osteoclast differentiation markers in osteoclasts derived from U937 cells upon stimulation with PMA and VD3. Analysis was performed by QRT-PCR. In each histogram, expression levels are expressed on the *y* axis as relative quantity (RQ), represented as mean ± S.E.M. The *x*-axis shows the different treatments: None (Ctr), Zoledronate (ZA), MgCl_2_ (Mg), or both ZA + Mg. Analyzed genes are listed at the top of each histogram.

**Figure 4:**
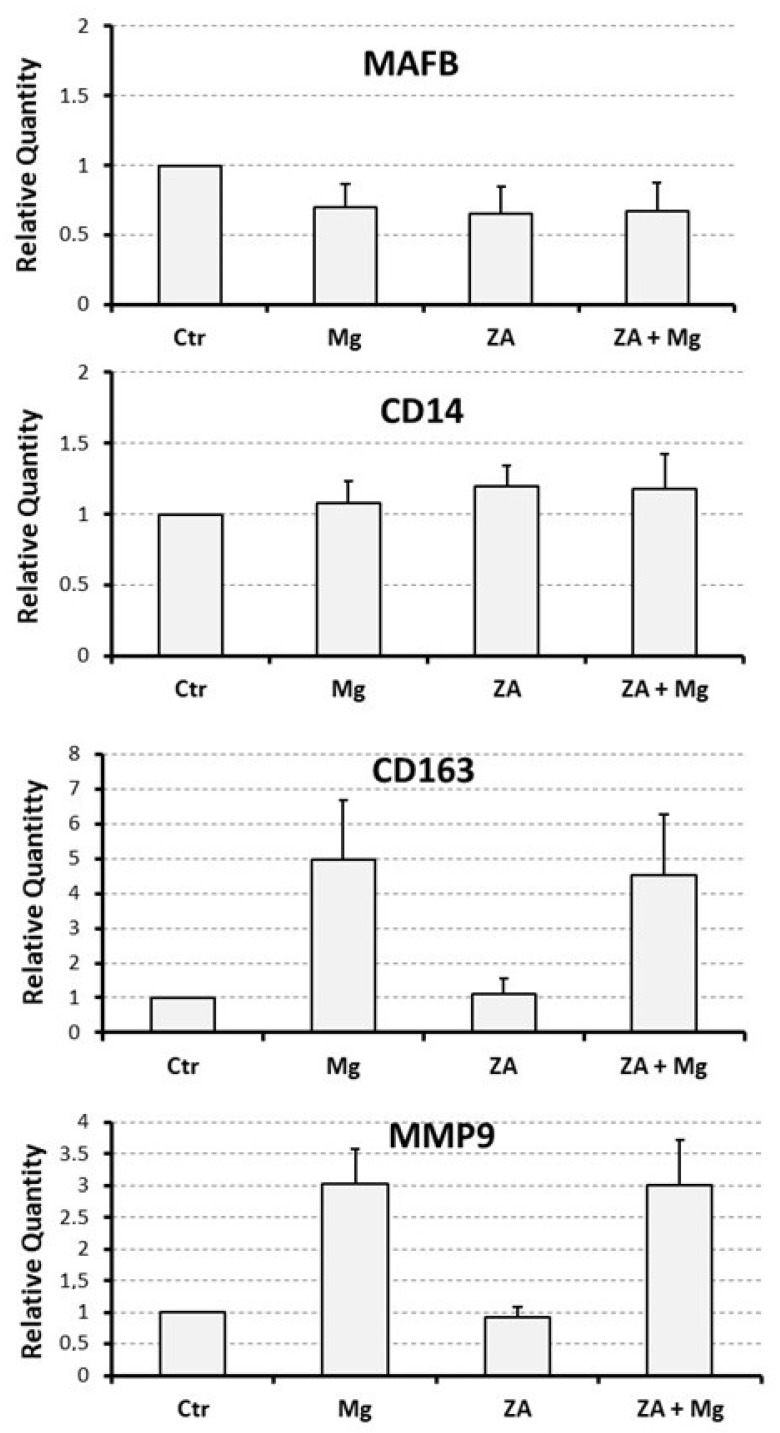
Effect determined by treatment with MgCl_2_ and ZA on the mRNA expression of monocyte/macrophage differentiation markers in osteoclasts derived from U937 cells upon stimulation with PMA and VD3. Analysis was performed by QRT-PCR. In each histogram, expression levels are expressed on the *y* axis as relative quantity (RQ), represented as mean ± S.E.M. The *x*-axis shows the different treatments: None (Ctr), Zoledronate (ZA), MgCl_2_ (Mg), or both (ZA + Mg). Analyzed genes are listed at the top of each histogram.

Molecular analysis was performed by QRT-PCR to evaluate the mRNA expression levels of a number of osteoclast and monocyte–macrophage differentiation markers [26,27,32,33,34,35]. The osteoclast markers included the following: NFATC1, the master regulator of osteoclast differentiation; DCST1, a surface antigen mediating the osteoclast cell fusion; ACP5, a phosphatase responsible for the degradation of the mineral component of the bone extra-cellular matrix; and CTSK and MMP9, two proteases responsible for the degradation of the protein component of the same matrix. The monocyte–macrophage markers included instead: MAFB, the master regulator of monocyte–macrophage differentiation; CD14, a surface antigen more related to monocytes and M1 activation; and CD163, a surface antigen more related to macrophages and M2 activation. The results of this analysis are presented in Figure 3 and Figure 4 as bar histograms, and in Table 1, as numerical values, always reporting the RQ values obtained by the various QRT-PCR reactions in terms of mean ± S.E.M. As expected, treatment with MgCl_2_ promoted upregulated mRNA expression of all the studied osteoclast differentiation markers. Among them, the most remarkable was represented by ACP5, exhibiting a 7.5 ± 1.4-fold increase of its transcript levels, whereas for the other genes of the same group the degree of mRNA induction was: 3.0 ± 0.6-fold for MMP9, 2.0 ± 0.3-fold for CTSK, 1.6 ± 0.1-fold for DCST1 and 1.3 ± 0.1-fold for NFATC1. Among the monocyte–macrophage markers, a similar result was observed for the mRNA expression of the CD163 antigen, undergoing a 5.0 ± 1.7-fold increase, whereas the transcript levels of the MAFB transcription factor and the CD14 antigen appeared to be only minimally affected by MgCl_2_ treatment. With the only exception being ACP5, presenting a 2.0 ± 0.3-fold increase of its mRNA expression, treatment with ZA was substantially unable to determine an appreciable effect on both the considered gene categories. Surprising data arose, on the contrary, from the combined treatment with ZA and MgCl_2_, disclosing a synergic effect on virtually all the analyzed osteoclast markers. The most striking result was, again, observed for ACP5, showing a 20.9 ± 4.0-fold upregulation of its mRNA expression, implying an about tripled effect in comparison with MgCl_2_ alone. The other genes of the same group confirmed this trend, although to a lesser extent. In fact, the entity of mRNA induction was: 3.2 ± 0.6-fold for CTSK, 2.1 ± 0.5-fold for DCST1, and 1.9 ± 0.1-fold for NFATC1, thus sensibly higher values as compared to those, already listed before, elicited by MgCl_2_ alone. In this regard, the MMP9 gene represented an exception, exhibiting identical values under the two considered treatment conditions (3.0 ± 0.7 versus 3.0 ± 0.6, respectively). The same effect was not observed on monocyte–macrophage markers, although it is worth underlining that the transcript levels of CD163 remained as high as those observed with MgCl_2_ alone (4.5 ± 1.7 versus 5.0 ± 1.7). Statistical analysis, conducted with the Anova procedure, results were highly significant for all osteoclast markers but one (DCST1) and for one out of the three monocyte–macrophage markers (CD163) (see Table 1, right column).

**Table 1:** Numerical values and statistical data of the molecular analysis performed by QRT-PCR on different osteoclast and monocyte–macrophage differentiation markers. The table shows the results of QRT-PCR already presented in Figure 3 and Figure 4, reported as values of relative quantity (RQ) and indicated as mean ± S.E.M. Comparisons were performed among treated (Mg, MgCl_2_; ZA, Zoledronate; ZA + Mg, combined treatment) and untreated (Ctr) osteoclasts derived from U937 cells upon stimulation with PMA and VD3. Statistical analysis was performed with the Anova procedure, reporting the relative results in the last column on the right. These results were considered significant when exhibiting *p* values ≤ 0.05 and highly significant when exhibiting *p* values ≤ 0.01.

Analyzed Marker	Ctr	Mg	ZA	ZA + Mg	Anova, *p* Value
NFATC1	1	1.3 ± 0.1	1.2 ± 0.1	1.9 ± 0.2	0.005
DCST1	1	1.6 ± 0.1	1.2 ± 0.3	2.1 ± 0.5	0.3
ACP5	1	7.5 ± 1.4	2.0 ± 0.3	20.9 ± 4.0	0.002
CTSK	1	2.0 ± 0.3	1.3 ± 0.3	3.2 ± 0.6	0.01
MMP9	1	3.0 ± 0.6	0.9 ± 0.2	3.0 ± 0.7	0.01
MAFB	1	0.7 ± 0.2	0.7 ± 0.2	0.7 ± 0.2	0.4
CD14	1	1.1 ± 0.2	1.2 ± 0.2	1.1 ± 0.3	0.8
CD163	1	5.0 ± 1.7	1.1 ± 0.4	4.5 ± 1.7	0.01

**Figure 5:**
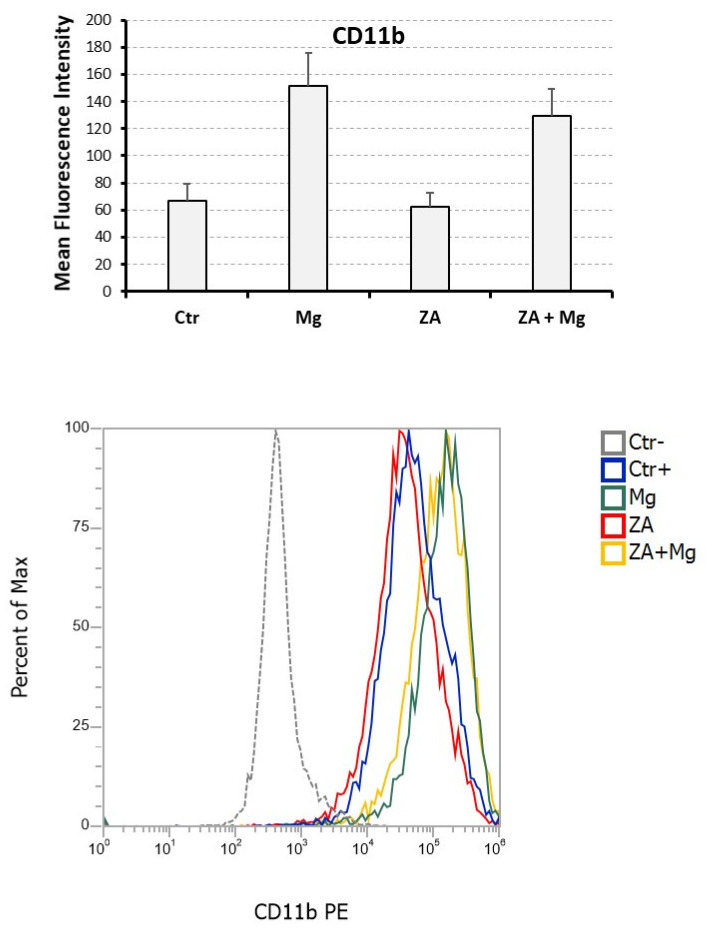
Flow cytometry analysis of the CD11b myeloid differentiation marker performed upon treatment with MgCl_2_, ZA, or both in osteoclasts derived from U937 cells by stimulation with PMA and VD3. Expression of the CD11b surface antigen was assessed by flow cytometry. The (**upper panel**) is a histogram showing, on the *y* axis, the mean fluorescent intensity (MFI) measured upon staining with an anti-CD11b PE antibody and indicated as mean ± S.E.M. The *x* axis shows the different treatments received by osteoclasts derived from U937 cells upon stimulation with PMA and VD3: None (Ctr), MgCl_2_ (Mg), Zoledronate (ZA) or both (ZA + Mg). The (**lower panel**) is a flow cytometry histogram of a representative experiment, in which the *y* axis shows the percentage of cells, while the *x* axis shows the MFI. The treatments are highlighted by different colors. Ctr- refers to undifferentiated U937 cells, that were not stimulated with PMA and VD3, while Ctr+ are differentiated U937 cells that were stimulated with PMA and VD3 but did not receive Mg or ZA treatment.

Immune phenotype analysis was carried out by flow cytometry assessment of the CD11b antigen [36], for which we estimated the positivity percentage and the mean fluorescence intensity (MFI) (Figure 5). In comparison with undifferentiated U937 cells, not stimulated with PMA and VD3, all samples receiving such stimulation exhibited a CD11b positivity higher than 95% (not shown) indicating that, at least for this parameter, the analyzed treatment conditions apparently did not affect the entity of osteoclast differentiation. In spite of this, treatment with MgCl_2_ resulted in a more than two-fold increase in CD11b MFI (151.8 ± 24.3 versus 66.8 ± 12.3 of untreated control cells), whereas ZA determined an almost 10% reduction in the same value when used alone (62.3 ± 10.7 versus, again, 66.8 ± 12.3 of untreated control cells) and an about 15% decrease when added to MgCl_2_ (129.5 ± 19.5 versus 151.8 ± 24.3 of MgCl_2_). Statistical analysis, conducted using the Anova procedure, attested that the differences observed among the compared MFI values were highly significant (*p* = 0.007).

**Figure 6:**
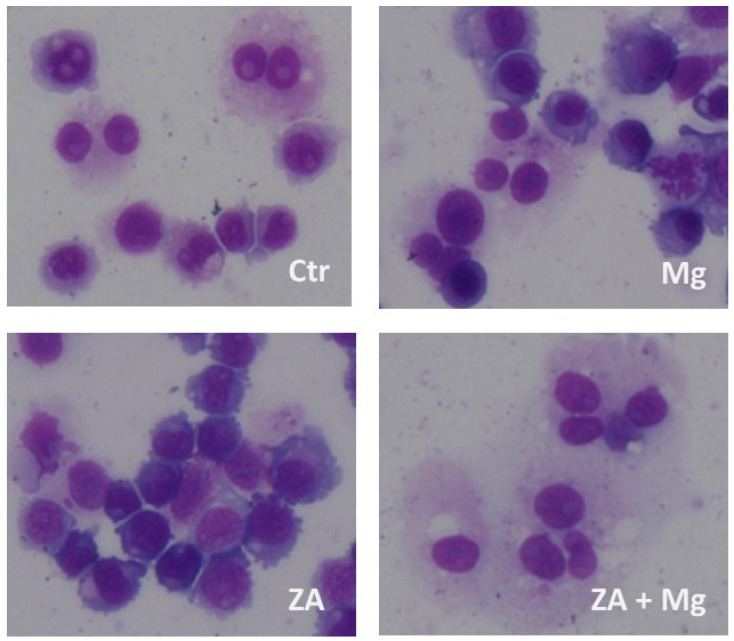
Morphological analysis performed upon treatment with MgCl_2_, ZA, or both in osteoclasts derived from U937 cells by stimulation with PMA and VD3. U937 cells, under the same experimental conditions of previous figures (Figure 3, Figure 4 and Figure 5), were subjected to cyto-centrifugation, May–Grunwald–Giemsa staining and microscope examination. The figure shows the results of a representative experiment. Ctr represents osteoclasts derived from U937 cells stimulated with PMA and VD3, that did not receive further treatments. Treated samples were instead exposed to MgCl_2_ (Mg), Zoledronate (ZA), or both (ZA + Mg).

Morphological analysis, performed on cyto-centrifuged samples upon staining with May–Grumwald–Giemsa, put in evidence that, as expected, 91% of the untreated control cells presented a morphologically differentiated phenotype, although osteoclasts belonging to this sample contained only one or two nuclei (Figure 6). Treatment with MgCl_2_ resulted in an apparently reduced percentage of cells with a terminally differentiated morphology (54%) but, in this case, osteoclasts contained three or four nuclei, testifying in any case a more differentiated phenotype than the control. Treatment with ZA was responsible for a dramatic reduction in fully mature cells (20%) that were also characterized by the complete absence of multi-nucleated osteoclasts. The simultaneous treatment with ZA and MgCl_2_ gave rise to a differentiation effect (88%) that was numerically similar to that of the control but with osteoclasts containing 5 to 10 nuclei.

The conclusions emerging from this part of the work are therefore that ZA alone exerts an inhibitory effect on osteoclast differentiation that we can define: absent, at the molecular level, weak, on immune phenotype, and strong, on morphology. The addition of MgCl_2_ to ZA treated cells determines not only a reversion of the ZA effect but even the appearance of a synergism between the two compounds, which became especially evident on the molecular and morphological phenotype.

## 4. Discussion

BPs are anti-resorptive drugs that, due their capacity to inhibit osteoclast activity, are used in several skeletal disorders all sharing the loss of bone tissue as major feature. Unfortunately, in addition to their therapeutic effect, these compounds can also provoke a serious side effect known as ONJ. The depletion of osteoclasts, responsible for a reduced ability to fight the bacterial invasion and to eliminate the infected bone, probably plays a crucial role in the development of this condition. Based on this premise we postulate that a local re-activation of osteoclast function might cure or even prevent ONJ.

Magnesium plays a key role in bone metabolism, where it regulates several functions, including osteoclast formation [37,38]. This effect is complex and concentration-dependent since osteoclasts are paradoxically stimulated by both a deficiency of Magnesium [39,40] or an excess of this element [26,41]. In a previous report, published by our research group, we have already proposed a likely biochemical mechanism that could underlie this apparently contradictory behavior of Magnesium [26]. The starting point of our work has been, in any case, represented by the observation that, when used at supra-physiological concentrations, Magnesium is a powerful inducer of osteoclast differentiation [26]. To assess whether this activity is also maintained in the presence of BPs, we have used an in vitro experimental model in which U937 cells, differentiated to osteoclasts through a sequential stimulation with PMA and VD3, have been concomitantly subjected to treatment with MgCl_2_ and/or ZA. The choice of this system is, in our opinion, a crucial aspect of the project and bases its biological rationale on the fact that, besides the physiological role played in the regulation of osteoclast differentiation [24], VD3 is also usually administered to patients under treatment with BPs [42,43]. As previously observed, MgCl_2_ determined a remarkable induction of osteoclast differentiation that was especially evident on molecular and immune phenotype. Morphological analysis revealed the presence of two cell subpopulations, one of which was represented by osteoclasts more differentiated than the control whereas the other consisted of undifferentiated cells still presenting a precursor aspect. This finding was consistent with the delay of proliferation that, in parallel, was observed on the same sample by flow cytometry analysis of the cell cycle. On the other hand, ZA strongly inhibited osteoclast differentiation at the morphological level, whereas this effect appeared only weakly exerted on immune phenotype and totally absent at the molecular level. These data indicate a post-translational block of osteoclast differentiation that was in perfect agreement with the universally accepted mechanism of action of ZA. In fact, according to the current view, ZA and other N-BPs act inhibiting the Farnesyl Pyro-Phosphate Synthase (FPPS) enzyme which is required to add a lipid anchor to a number of G proteins promoting, in addition to other biological functions, the cytoskeleton arrangements and morphological changes that are necessary to accomplish terminal differentiation [6]. Co-treatment with ZA and MgCl_2_ resulted in a quite unexpected situation since, not only was ZA unable to inhibit the powerful differentiation effect of MgCl_2_ but, on the contrary, this combination gave rise to a powerful synergistic effect on osteoclast differentiation that was especially evident at the molecular and morphological level. This conclusion was, in fact, respectively, testified by the over 20-fold increase in ACP5 mRNA expression and the massive formation of giant multi-nucleated osteoclasts containing 5 to 10 nuclei, implying that the presence of ZA amplified by about three times the osteoclast differentiation effect of MgCl_2_. The molecular mechanism by which MgCl_2_ favors osteoclast differentiation and other biological functions, is complex and poorly understood. What is known is that the Mg^2+^ cation binds to ATP^4−^ forming MgATP^2−^ that in turn activates about three hundred enzymes comprising protein kinases and ATPases regulating a huge number of cell processes [26]. A similar operation is mediated by Mg^2+^, through a binding activity with GTP on GTPases. It is therefore possible that osteoclast differentiation is one of the cell processes regulated by this modality. An additional issue that remains to be elucidated is represented by the molecular mechanism by which ZA potentiates, rather than the opposite, the osteoclast differentiation effect of MgCl_2_. A possible explanation, in this case, could rely in the fact that the interaction of both PP and N-BPs with the active catalytic site of the FPPS enzyme is mediated by the Mg^2+^ cation [44]. It is therefore possible that, perhaps acting through a conformational change mechanism, an increase in Mg^2+^ concentration might determine an opposite effect on the recruitment of PP and N-BP, favoring the former and inhibiting the latter. However, this hypothesis can only partly explain our data because, as far as is known, the FPPS enzyme is devoid of transcriptional effects that are instead clearly observed in U937-derived osteoclasts subjected to co-treatment with ZA and MgCl_2_. An aspect deserving some attention is, in our opinion, that a couple of the analyzed genes, in particular MMP9 and CD163, although clearly induced by MgCl_2_, did not undergo a further upregulation upon ZA addition but maintained a high mRNA expression level. Interestingly, both these antigens are M2 macrophage polarization markers [45]. Several authors have suggested that osteoclast phenotype is very close to that of M1 polarized macrophages [46,47]. An interpretation of our data might be that MgCl_2_ favors the differentiation of variant osteoclasts, characterized by an M2 phenotype, rendering them different from the typical osteoclasts, exhibiting instead a classical M1 phenotype. The presence of VD3, often considered an M2 polarizing agent [48], could perhaps contribute to the appearance of this phenotype. These considerations, in light of the involvement of M2 polarized macrophages in post-inflammatory tissue repair, open an interesting question regarding their possible consequences on ONJ healing.

To conclude, our finding suggests that, in various forms (solution, gel, foam, membrane, powder), MgCl_2_ or other salts, or more generically other compounds containing Magnesium, might be used in a topical therapy to cure or prevent ONJ. This approach would allow patients to continue the systemic therapy with BPs, thus maintaining its therapeutic benefit on the skeletal sites interested by the cured disease, counteracting on the contrary its effect exclusively in the oral cavity, where a local treatment can be easily administered. Such a treatment could, for example, be applied to an area of exposed bone, to cure an already established ONJ, or to the alveolar/peri-alveolar bone soon after a tooth extraction, to prevent the onset of a new ONJ. It is worth underlining that, according to our data, the best efficacy of this strategy is expected in patients under treatment with ZA, or probably other BPs, although a therapeutic effect is in any case expected in their absence considering the capacity of MgCl_2_, clearly demonstrated in this report, to induce osteoclast differentiation even when used alone.

To confirm the hypothesis under discussion, further investigation is, of course, necessary both in vitro and in vivo. To begin, a first aspect to clarify in vitro would be, for example, how MgCl_2_ interacts with other BPs or even non-BP anti-osteoclast drugs, such as Denosumab. To continue, our finding, obtained in a VD3-dependent system, should be verified in a different and Rankl-dependent context [49]. The data obtained meanwhile, should be also validated on osteoclasts deriving from normal primary monocytes [50]. The real biological activity of the various osteoclast categories described so far should be then assessed with an appropriate functional test such as bone resorption assay [25]. Finally, our observation should be confirmed in vivo using an ONJ animal model [51,52,53,54] and patients affected by ONJ or presenting a high risk to develop this condition [23]. Therefore, a long and complex experimental process is still needed to provide a definitive demonstration of the clinical applicability of an MgCl_2_-based topical therapy in ONJ.

## 5. Conclusions

The conclusions of our work are that, in addition to their capacity to induce osteoclast apoptosis, BPs are also characterized by a strong inhibition activity on osteoclast differentiation. This effect is not only abrogated by MgCl_2_ but even transformed, through a sort of paradox effect, in an opposite and striking potentiation of osteoclast differentiation. In our opinion, these results open the possibility to use MgCl_2_ as a topic agent to cure or prevent ONJ; although, this circumstance needs, of course, to be properly confirmed by further studies in vitro and in vivo.

## 6. Patents

The results presented in this manuscript have been previously used to file an Italian patent entitled “Therapy of Osteonecrosis of the Jaw”, Inventors Alexis Grande, Patent Status Pending, Priority Number 102023000002301, Priority Date 10/02/2023, License International, Commercial Rights Exclusive, Availability Available; viewable at the link https://www.knowledge-share.eu/en/patent/therapy-of-osteonecrosis-of-the-jaw/ (accessed on 9 March 2023).

## Data Availability

The data presented in this study are available on request from the corresponding author.
